# Management of Diabetes Mellitus: Could Simultaneous Targeting of Hyperglycemia and Oxidative Stress Be a Better Panacea?

**DOI:** 10.3390/ijms13032965

**Published:** 2012-03-06

**Authors:** Omotayo O. Erejuwa

**Affiliations:** Department of Pharmacology, School of Medical Sciences, Universiti Sains Malaysia, 16150 Kubang Kerian, Kelantan, Malaysia; E-Mail: erejuwa@gmail.com; Tel.: +60-9766-6877; Fax: +60-9765-3370

**Keywords:** hyperglycemia, intensive therapy, glycemic control, hypoglycemic drugs, oxidative stress, antioxidants

## Abstract

The primary aim of the current management of diabetes mellitus is to achieve and/or maintain a glycated hemoglobin level of ≤6.5%. However, recent evidence indicates that intensive treatment of hyperglycemia is characterized by increased weight gain, severe hypoglycemia and higher mortality. Besides, evidence suggests that it is difficult to achieve and/or maintain optimal glycemic control in many diabetic patients; and that the benefits of intensively-treated hyperglycemia are restricted to microvascular complications only. In view of these adverse effects and limitations of intensive treatment of hyperglycemia in preventing diabetic complications, which is linked to oxidative stress, this commentary proposes a hypothesis that “simultaneous targeting of hyperglycemia and oxidative stress” could be more effective than “intensive treatment of hyperglycemia” in the management of diabetes mellitus.

Diabetes mellitus is a metabolic disorder characterized by chronic hyperglycemia with impaired metabolism of carbohydrate, fat and protein resulting from defects in insulin secretion or insulin action or both [1]. The primary aim of the current management of this disorder is to achieve and/or maintain the recommended optimal glycemic goal (glycated hemoglobin level of ≤6.5%) [1], which is beneficial in both type 1 and type 2 diabetes mellitus [2,3]. However, recent evidence indicates that intensive treatment of hyperglycemia is associated with increased weight gain, severe hypoglycemia and higher mortality [4–6]. Besides, most of the benefits of intensive therapy of hyperglycemia are limited to microvascular complications [2,3,5]. Evidence also indicates that multiple drugs are required to achieve optimal glycemic target in many diabetic patients [7]. In fact, in many diabetic patients in whom optimal glycemic goal is achieved, glycemic control deteriorates even with optimal drug therapy [8]. It does suggest that with the current hypoglycemic or antidiabetic drugs, it is difficult to achieve and/or maintain tight glycemic control in diabetic patients [7,8]. In many developing countries, the vast majority of diabetic patients have limited or lack access to quality healthcare providers and good therapeutic monitoring. All these may contribute to the unabated increase in global prevalence of diabetes mellitus and its complications [9,10].

While increased weight gain could be due to some component drugs (such as sulphonylureas or insulin) of the intensive therapy regimens, hypoglycemia could be drug-induced or comorbidity-induced [4–6,11]. Considering the evidence that associates hypoglycemia with increased mortality [4–6], higher incidence of mortality in intensive therapy group could be due to hypoglycemia or too low levels of glycosylated hemoglobin [4–6,11]. However, it is difficult to contend that increased mortality was entirely due to hypoglycemia. The possibility of drug-induced or drug-associated toxicities could not be ruled out. For instance, rosiglitazone, which has been prohibited and withdrawn from the market in Europe, was one of the hypoglycemic drugs used to achieve intensive therapy of hyperglycemia in Action to Control Cardiovascular Risk in Diabetes (ACCORD) [5]. If these findings are anything to go by, does it not suggest that targeting hyperglycemia as the only therapeutic goal in the management of diabetes mellitus could be detrimental to diabetic patients? In addition, the current hypoglycemic drugs are characterized by limitations and adverse effects [4–6]. Together with the limitations of intensive glycemic treatment (only beneficial in reducing the risk of microvascular complications, but not macrovascular disease complications) [2,3,5], does it not imply that targeting hyperglycemia alone is not only deleterious but also limited and ineffective? The latest figures predict that the global incidence of diabetes mellitus, which was estimated to be 366 million in 2011, will rise to 522 million by 2030 [10]. In view of these frightening statistics on the prevalence of diabetes mellitus [10] and on the lack of adequate healthcare [9], together with the associated diabetic complications, morbidity and mortality [2–6,11], does it not suggest that there is an urgent need for a better therapeutic management of this disorder? Taken together, with these findings and statistics, it can be contended that it is high time alternative and/or complementary therapies to the currently available hypoglycemic agents (which target primarily hyperglycemia only) were sought.

Currently, one of such complementary options is the potential of “concurrently targeting hyperglycemia and oxidative stress”. Oxidative stress is defined as an “imbalance between oxidants and antioxidants in favor of the oxidants, potentially leading to damage” [12]. It is implicated in the pathogenesis and complications of diabetes mellitus. The role of oxidative stress is more definite in the pathogenesis of type 2 diabetes mellitus than in type 1 diabetes mellitus [13]. In regard to diabetic complications, there is compelling evidence in support of the role of oxidative stress in both types of diabetes mellitus [14]. Evidence suggests that elevated reactive oxygen species (ROS), which causes oxidative stress, accumulate in certain micro milieu or tissues (such as retina and kidney) where they cause damage or toxicity [14]. In diabetes mellitus, oxidative stress is enhanced through various sources such as hyperglycemia, dyslipidemia, hyperinsulinemia, insulin resistance, impaired antioxidant defense network, uncoupling of ROS-generating enzymes, elevated level of leptin and sedentary lifestyle [15]. A number of mechanisms or pathways by which hyperglycemia, the major contributing factor of increased ROS production, causes tissue damage or diabetic complications have been identified [14]. These include: hyperglycemia-enhanced polyol pathway; hyperglycemia-enhanced formation of advanced glycation endproducts (AGEs); hyperglycemia-activated protein kinase C (PKC) pathway; hyperglycemia-enhanced hexosamine pathway; and hyperglycemia-activated Poly-ADP ribose polymerase (PARP) pathway [14]. These pathways are activated or enhanced by hyperglycemia-driven mitochondrial superoxide overproduction [14].

Even though oxidative stress plays an important role in its pathogenesis and complications, unlike other diseases characterized by oxidative stress, diabetes mellitus is unique. Its cure (restoration of euglycemia, e.g., via pancreas transplants) does not prevent oxidative stress and diabetic complications [16–18]. This is very important because hyperglycemia exacerbates oxidative stress which is linked to diabetic complications [14,18]. Theoretically, restoration of euglycemia should prevent oxidative stress and diabetic complications. However, this is not the case [16–18]. At present, it remains unclear why restoration of euglycemia does not automatically prevent oxidative stress and diabetic complications. Phrases such as “hyperglycemic memory”, “glycemic memory”, “metabolic memory”, or simply “lasting memory” have been introduced to describe this difficult-to-explain observation or phenomenon [14,18]. The phrase or term refers to the development of diabetes-related complications (both microvascular and macrovascular) in diabetic patients after normoglycemia has been restored [14,18,19]. It is a phenomenon whereby previous hyperglycemic milieu is remembered in many target organs such as heart, eyes, kidneys and nerves [14,18,19]. This phenomenon is also documented in diabetic animals [16,17]. Compelling evidence implicates the role of oxidative stress as an important mechanism by which glycemic memory causes tissue damage and diabetic complications [14,18,19].

Considering that intensive therapy of hyperglycemia is associated with increased weight gain, severe hypoglycemia and higher mortality than standard therapy of hyperglycemia [4–6,11], theoretically, discontinuation of intensive therapy should reduce or minimize these adverse effects. However, in view of higher incidence of diabetic complications (of which oxidative stress plays an important role) in conventionally-treated diabetic patients [2,3], targeting oxidative stress in these patients might be beneficial. In other words, it is possible that the combination of a conventional therapy of hyperglycemia and antioxidant therapy might be more effective and beneficial than intensive therapy of hyperglycemia alone, which is the gold standard at the moment. Besides, this combination might also be devoid of the intensive therapy-associated adverse effects (increased weight gain, severe hypoglycemia and higher mortality) [4–6,11]. At this juncture, the question that arises is: “is there evidence in support of such a hypothesis?” At the moment, there are no findings in large-scale clinical trials that corroborate this hypothesis. However, there are experimental and clinical data, though limited, that suggest simultaneous target of hyperglycemia and oxidative stress might be a better therapeutic option in the management of diabetes mellitus.

A study that targeted hyperglycemia and oxidative stress with insulin and antioxidants (vitamin E and C), respectively in diabetic rats found that the combination of these two classes of agents normalized hyperglycemia, weight loss and oxidative stress parameters more considerably than either agent [20]. Similarly, in diabetic rats, targeting hyperglycemia and oxidative stress with glibenclamide or metformin and antioxidant (honey), respectively improved glycemic control, lipid profile and renal function [21]. Likewise, simultaneous target of hyperglycemia and oxidative stress ameliorated oxidative stress in pancreas [22,23] and kidney [24] of diabetic rats more considerably than targeting hyperglycemia alone. Additional evidence in support of this hypothesis comes from a study by Qiang and co-workers [25]. The authors found that neither gliclazide (a free radical scavenger) nor glibenclamide had an effect on glycemia in both non-diabetic and diabetic rats [25]. However, the study indicated gliclazide (but not glibenclamide) was beneficial in ameliorating oxidative stress and related parameters [25]. The study also found that gliclazide but not glibenclamide prevented reduced myelinated fiber area, increased fiber density and decreased axon/myelin ratio in diabetic rats [25]. This study clearly shows that targeting oxidative stress, irrespective of the hyperglycemia, prevents peripheral neuropathy in diabetic rats [25].

In humans with type 1 or type 2 diabetes mellitus, it was reported that maintenance of optimal glycemic control alone was less effective in ameliorating oxidative stress compared to when optimal glycemic control was combined with antioxidant (vitamin E) therapy [26,27]. A study showed that treatment of type 2 diabetic patients with gliclazide ameliorated oxidative stress compared with baseline and the glibenclamide-treated diabetic patients [28]. Interestingly, the levels of blood glucose and glycosylated hemoglobin were elevated and similar in both groups. It thus indicates that oxidative stress can be controlled irrespective of hyperglycemia [28]. This study suggests that an antioxidant or a free radical scavenger, such as gliclazide, combined with conventional therapy of hyperglycemia could be beneficial in diabetic patients. Another study by Ceriello and colleagues also demonstrated the beneficial effects of targeting hyperglycemia and oxidative stress simultaneously using insulin and antioxidant (vitamin C), respectively on oxidative stress and endothelial dysfunction [29]. The authors reported that neither insulin treatment (despite normalization of hyperglycemia) nor antioxidant therapy (vitamin C) was able to sufficiently ameliorate oxidative stress or normalize endothelial dysfunction [29]. On the contrary, combination of the two agents considerably and adequately ameliorated oxidative stress and normalized endothelial dysfunction in type 1 diabetic patients [29].

By and large, these studies indicate that co-administration of oral hypoglycemic drugs (and/or insulin) and antioxidants (honey or vitamins C and E) does not reduce or interfere with the hypoglycemic or antioxidant effect of either agent. Therefore, a combination of these two classes of agents is justifiable in the management of diabetes mellitus. With the combination, hypoglycemic drugs will target hyperglycemia to improve glycemic control. In view of evidence which implicates hyperglycemia as the sole contributor of ROS and oxidative stress in diabetes mellitus, targeting hyperglycemia may also be beneficial in reducing oxidative stress [28]. On the other hand, antioxidant therapy will help to ameliorate oxidative stress. Besides, some antioxidants have hypoglycemic effect and improve glycemic control [21,30,31]. As demonstrated in some of those studies, antioxidant therapy has a potential to ameliorate oxidative stress in different compartments including the blood vessels and tissues which are targets of oxidative stress and diabetic complications. These include, but are not limited to, retina [16,17], pancreas [22,23], kidney [24,30], nerves [25], vasculature [29] and erythrocytes [31]. Considering the potential limitations of antioxidant therapy, other therapeutic manipulations of oxidative stress such as mitochondria-targeted antioxidants, inhibition of the mitochondrial ROS overproduction, or inhibition of hyperglycemia-activated pathogenic and signaling pathways are viable complementary options. Any of these could be combined with hypoglycemic agents.

Besides, targeting hyperglycemia and oxidative stress may also offer additional benefits. These include amelioration of oxidative stress and damage in the pancreas [22,23,32]. In the case of pancreas, antioxidant therapy may help to reduce glucotoxicity (mediated via oxidative stress), prevent deterioration of beta-cell function and improve glycemic control [33,34]. Targeting hyperglycemia and oxidative stress may help to ameliorate lipid abnormalities (e.g., elevated low-density lipoproteins (LDLs)) and improve oxidative stress [21,35], which increases the susceptibility of LDLs to oxidation and glycation. This may prevent atherosclerosis and endothelial dysfunction. Antioxidants have further been shown to improve insulin resistance, insulin levels and C-peptide in diabetic rats [21,36,37]. Targeting hyperglycemia and oxidative stress may help to ameliorate intestinal oxidative stress and increase the bioavailability of essential macronutrients or co-administered antidiabetic agents [38,39]. Furthermore, antioxidants may help to ameliorate liver damage and hepatic oxidative stress which are common in diabetes mellitus [40,41]. Considering the roles of the liver in glucose homeostasis and in mediating the hypoglycemic effect of antidiabetic agents such as glibenclamide, antioxidant therapy may improve liver function and contribute to improved glycemic control. Moreover, co-administration of hypoglycemic drugs with antioxidants may help to prevent weight gain commonly observed with hypoglycemic drugs, especially glibenclamide [42]. This may be advantageous in obese or overweight type 2 diabetic patients. Moreover, since many diabetic patients usually develop hypertension which is also associated with oxidative stress, antioxidants may help to prevent or delay the development of hypertension and diabetic complications [43,44]. In addition, targeting hyperglycemia and oxidative stress concurrently may require lower doses of antidiabetic drugs, which in turn may help to minimize the adverse effects or toxicities of these agents.

In a nutshell, in view of the compelling evidence that demonstrates an association between an intensive therapy of hyperglycemia, it would be worthwhile to reconsider any therapeutic goal that target “hyperglycemia or glycemic control alone” in diabetic patients. Instead, targeting hyperglycemia and oxidative stress simultaneously might be a better option. While this commentary does not underestimate the importance of optimal glycemic control, however, the data, views and explanations presented or posited, do strengthen the hypothesis that the prospect of managing diabetes mellitus more effectively by targeting hyperglycemia and oxidative stress concurrently holds much promise. This hypothesis is worth investigating in diabetic patients. Therefore, both small and large randomized clinical trials that investigate the effects of targeting hyperglycemia and oxidative stress simultaneously in patients with type 1 or type 2 diabetes mellitus are highly desirable. Also, clinical trials that compare the effects of a standard or conventional therapy of hyperglycemia and antioxidant therapy with those of intensive therapy of hyperglycemia alone are also recommended. These clinical trials would help to substantiate or perhaps refute this hypothesis in diabetic human subjects.
